# Medicine in Government

**Published:** 1899-01-07

**Authors:** 


### Medicine in Government.

The very high distinction of the Grand Cross of the Order of the Bath, conferred upon Sir Hugh Owen, the late Permanent Secretary of the Local Government Board, on his retirement, has not only the effect of showing the great value placed by the Government upon his past services, but of showing also that, in their estimation, his office ranks in importance with that of the Clerk to the Privy Council, the same distinction in the same circumstances having fallen to Sir Charles Lennox Peel. All who know how much the efficiency of a public office depends upon its permanent head, as distinguished from the politicians by whom it may from time to time be represented in Parliament, will recognise that the Local Government Board itself may almost be regarded as a creation of the two Sir Hughs—father and son—who have directed its work for more than sixty years. The father retired in 1872 in order to devote himself to the improvement of education in the Principality; and his mantle in due time descended upon his son. Of the growth of their official duties and responsibilities in the years covered by their successive periods of activity the increasing bulk and increasing complexity of the annual reports are of themselves sufficient to show. They are also sufficient to suggest the inquiry whether this growth has not now exceeded the limits within which the attainment of the highest efficiency must always be confined, and whether, therefore, the time has not come at which some new arrangement would be alike practicable and desirable.

The portion of the duties devolving upon the Board which would seem to be most readily separable from the rest, and which, by reason of its importance, has the principal claim to be erected into an independent department, is unquestionably that which has relation to the public health, and to the numerous and increasing channels through which medical science may be brought to bear in he promotion of the welfare of the community. It may probably be assumed that the Medical Department of the Local Government Board, under the sagacious management of Sir Richard Thorne-Thorne, as under that of his distinguished predecessors, Sir John Simon and Sir George Buchanan, has enjoyed, and enjoys, a large share of independence; for the simple reason that its proceedings cannot be submitted to skilled criticism by any but a medical man, and that neither Sir Hugh Owen in the past, nor those who may succeed him in the future, would be likely to look upon the proposals of the Chief Medical Officer with any other feeling than respect. But experience, even very recent experience, has shown that the Medical Department, as a single branch of an office overworked in other directions, is not adequately represented in Parliament; and that the President of the Local Government Board, however desirous he may be to do his duty, cannot devote more than a small portion of his time to endeavours so to understand medical questions as to be able to present them to his colleagues in the Government, or to the House of Commons, with such simplicity as to render them clear of comprehension, and with force adequate to their importance. It may be laid down, as a general principle, that few men, and no politicians, can be expected to possess what is familiarly called "backbone" in relation to any subject of debate on the merits of which they are but imperfectly informed; and there is no description of law-making or of administration in which backbone is more imperatively required than in all matters connected with the applications of biology to the government of mankind. Public health not only demands a Minister, but a Minister of Cabinet rank, able to obtain from his colleagues full consideration of his views. As Lord Beaconsfield long ago had the penetration to see, the subject is of overwhelming national importance; and, although there are manifest objections to a Cabinet unduly large, yet the inevitably increasing complexity of public interests must not be sacrificed to the mere preservation of compactness.

